# The miR-146b-3p/TNFAIP2 axis regulates cell differentiation in acute myeloid leukaemia

**DOI:** 10.18632/aging.205441

**Published:** 2024-01-24

**Authors:** Gaochen Lan, Xiaolong Wu, Aiyue Zhao, Jinjian Lan, Qiusheng Guo, Bolin Wang, Fenglin Shen, Xiaoling Yu, Yanna Zhao, Ruilan Gao, Tianwen Xu

**Affiliations:** 1 Department of Oncology, The Second Affiliated Hospital of Fujian Medical University, Quanzhou, China; 2 College of Life Science, Zhejiang Chinese Medical University, Hangzhou, China; 3 The First Clinical College, Zhejiang Chinese Medical University, Hangzhou, China; 4 Department of Oncology, Affiliated Jinhua Hospital, Zhejiang University School of Medicine, Jinhua, China; 5 Institute of Hematology, The First Affiliated Hospital of Zhejiang Chinese Medical University, Hangzhou, China

**Keywords:** differentiation, TNFAIP2, miR-146b-3p, AML, mechanism

## Abstract

Our purpose is to verify that miR-146b-3p targets the downstream transcript TNFAIP2 in order to reveal the machinery underlying the miR-146b-3p/TNFAIP2 axis regulating acute myeloid leukaemia (AML) differentiation. Bioinformatics analyses were performed using multiple databases and R packages. The CD11b+ and CD14+ cell frequencies were detected using flow cytometry and immunofluorescence staining. The TNFAIP2 protein expression was evaluated using western blotting, immunocytochemistry and immunofluorescence staining. The qRT-PCR was conducted to detect the expression of TNFAIP2 and miR-146b-3p. TNFAIP2 and its correlated genes were enriched in multiple cell differentiation pathways. TNFAIP2 was upregulated upon leukaemic cell differentiation. miR-146b-3p directly targeted TNFAIP2, resulting in a decrease in TNFAIP2 expression. Forced expression of TNFAIP2 or knockdown of miR-146b-3p significantly induced the differentiation of MOLM-13 cells. In this study, we demonstrated that TNFAIP2 is a critical driver in inducing differentiation and that the miR-146b-3p/TNFAIP2 axis involves in regulating cell differentiation in AML.

## INTRODUCTION

Acute myeloid leukaemia (AML) refers to a group of blood-forming malignancies that are derived from malignant clones of haematopoietic stem/progenitor cells and characterized by proliferation of bone marrow blasts, inhibition of normal haematopoiesis, and blockade of differentiation [1]. The annual incidence of AML is 0.4 to 2.8 per 100,000, and AML accounts for approximately 40% of leukaemia-related deaths [2–4].

AML is a highly heterogeneous disease with complicated gene regulatory networks. TNF-alpha induced protein 2 (TNFAIP2) is relevant to the initiation of AML [5]. TNFAIP2 participates in multiple biological functions, including cell differentiation [5–8]. TNFAIP2 is highly expressed in bone marrow haematopoietic stem/progenitor cells and peripheral blood monocytes [7, 9]. Some studies showed that TNFAIP2 could be involved in RA signalling; thus, it could be considered a potential target gene for the therapeutic induction of cell differentiation in acute promyelocytic leukaemia [7, 10, 11]. Some studies reported that TNFAIP2 expression is upregulated during U937 cell differentiation, indicating that it has a close association with promoting leukaemic cell differentiation [10]. Although these studies have provided us with insight into the connection among differential expression of TNFAIP2, cell differentiation and the treatment of leukaemia, whether TNFAIP2 can directly induce leukaemic cell differentiation is not fully clarified, and the mechanism is still unclear.

Currently, the competing endogenous RNA (ceRNA) postulate yielded emerging perspectives for leukaemia investigation [12]. This hypothesis holds that miRNAs inhibit mRNA transcription or protein translation [13, 14]. miRNAs constitute a class of non-coding RNAs that are approximately 20-25 nucleotides in length [15]. The main functions of miRNAs include regulating growth and development, haematopoietic differentiation, organ formation, cell proliferation, apoptosis and metabolism, etc. [16]. A miRNA combines with the 3’ untranslated region (3’UTR) of a targeted mRNA, leading to decreased translation of the protein encoded by the target gene through inhibition of target gene expression or posttranslational modification [17].

Using the TargetScan (https://www.targetscan.org/vert_80/) bioinformatics prediction database, we found that many miRNAs can directly target TNFAIP2. In addition, some miRNAs were downregulated upon monocytic leukaemia cell differentiation. We determined the overlap between these two gene sets and identified miR-146b-3p. Therefore, we hypothesised that miR-146b-3p directly targets TNFAIP2, thus regulating AML cell differentiation. miR-146b-3p targets NF2, MAP3K10, HPGD, FAM107A, etc. mRNAs, resulting in tumorigenesis and progression in multiple malignant diseases [18–21]. Accumulating evidence has shown that miR-146b-3p participates in cell differentiation. miR-146b-3p inhibits MDFIC mRNA, thus blocking myoblast differentiation [22]. miR-146b-3p regulates differentiation and iodine uptake through the miR-146b-3p/PAX8/NIS axis [23]. In view of these observations, we hypothesized that the miR-146b-3p/TNFAIP2 axis is a possible mechanism by which differentiation is induced in AML cells.

## RESULTS

### TNFAIP2 expression was downregulated in AML patients

**Figure 1:**
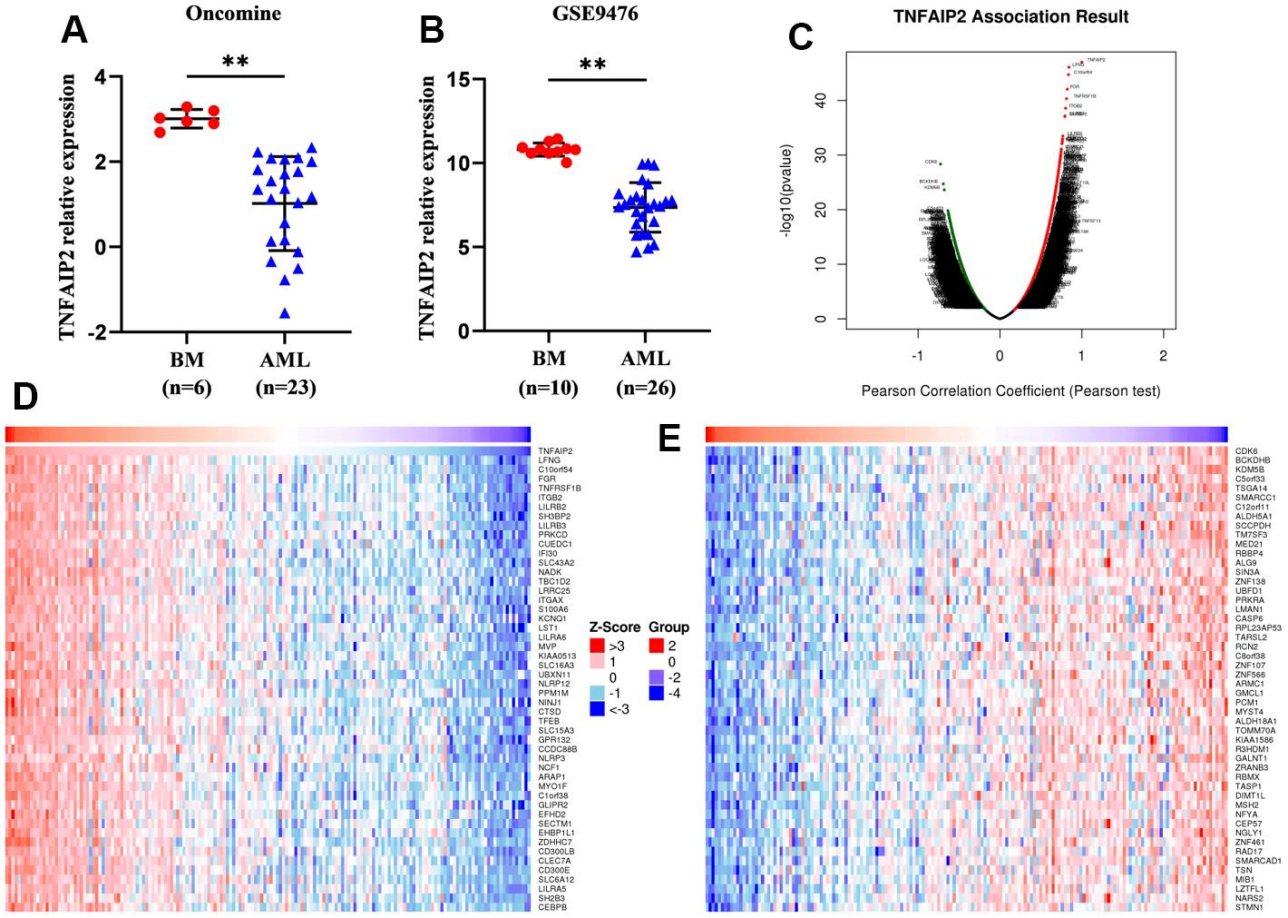
**Differentially expressed genes and TNFAIP2-correlated genes in AML patients.** (**A**) The transcript level of TNFAIP2 was significantly decreased in AML samples compared with normal samples in the Oncomine database. (**B**) The expression of TNFAIP2 mRNA was markedly decreased in AML samples compared with normal samples in the GSE9476 dataset. (**C**) Volcano plot of TNFAIP2-correlated genes. (**D**) Heatmap of the top 50 genes positively correlated with TNFAIP2, based on correlation coefficients. (**E**) Heatmap of the top 50 genes negatively correlated with TNFAIP2, based on correlation coefficients. BM: normal bone marrow samples. Normal distribution, t test, ** *P* < 0.01.

Using the GSE9476 dataset and Oncomine database, we analysed differentially expressed TNFAIP2 transcript among AML patients and healthy individual samples. The value of TNFAIP2 expression in AML samples was 7.36±1.44, markedly lower than the value of 10.81±0.37 in normal samples (*P* < 0.05, Figure 1B). A similar result was achieved in the analysis of the Oncomine database (*P* < 0.05, Figure 1A).

### Identification of the 100 genes most associated with TNFAIP2

We used the LinkedOmics database and Pearson correlation analysis to analyse the correlations between TNFAIP2 and other genes. The top 100 genes correlated with TNFAIP2 were obtained; these genes included LFNG, C10orf54, FGR, TNFRSF1B, and ITGB2, which were positively correlated with TNFAIP2, and CDK6, BCKDHB, KDM5B, SCCPDH, and C5orf33, which were negatively correlated with TNFAIP2 (*P* < 0.05, Figure 1C–1E).

### Enrichment analysis of TNFAIP2-correlated genes

**Figure 2:**
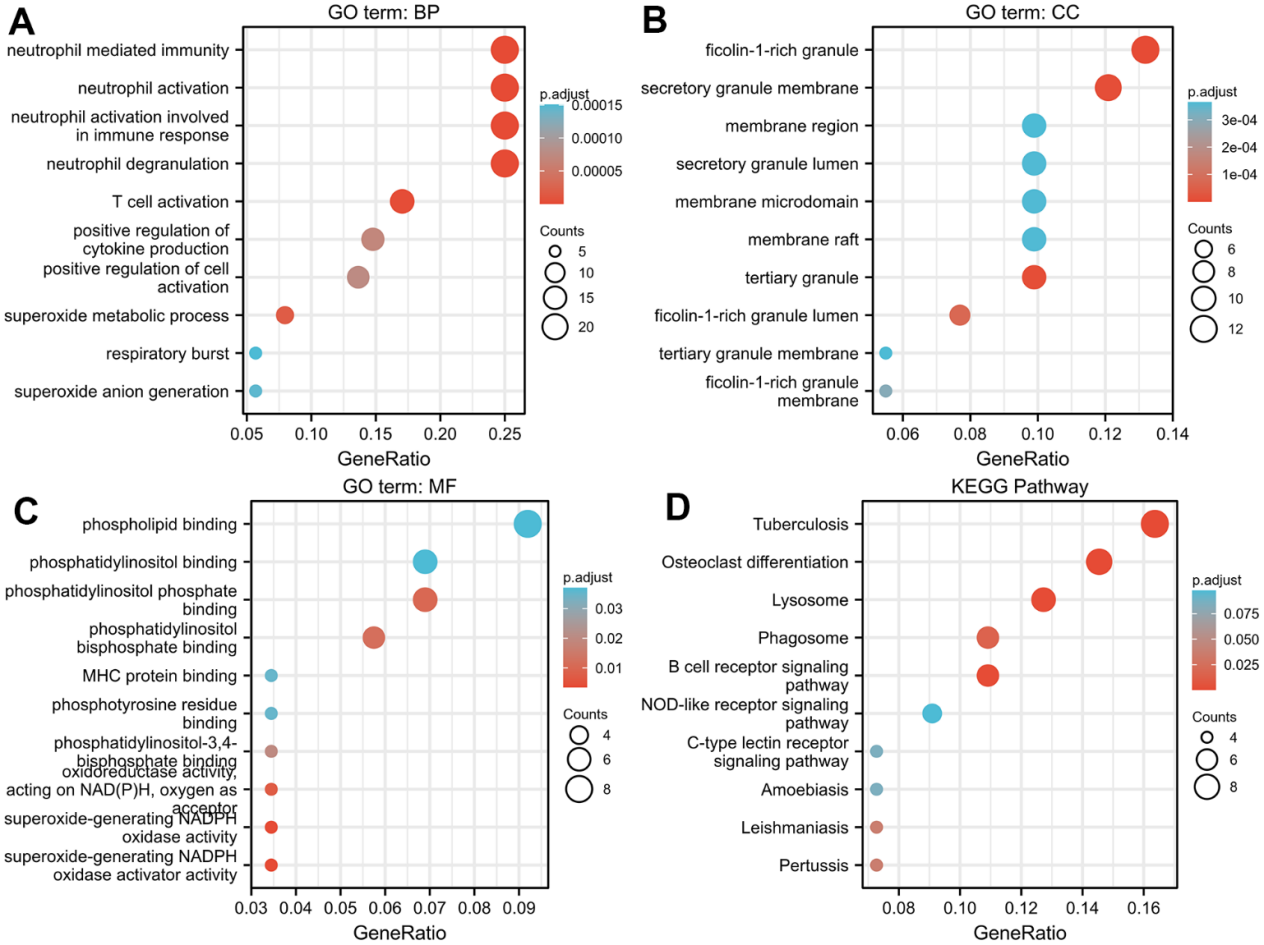
**GO/KEGG enrichment analysis of genes correlated with TNFAIP2 in AML patients.** (**A**) The GO biological process functional enrichment analysis of TNFAIP2-correlated genes. (**B**) GO cellular component functional enrichment analysis of TNFAIP2-correlated genes. (**C**) GO molecular function functional enrichment analysis of TNFAIP2-correlated genes. (**D**) KEGG pathway enrichment analysis of genes correlated with TNFAIP2.

**Figure 3:**
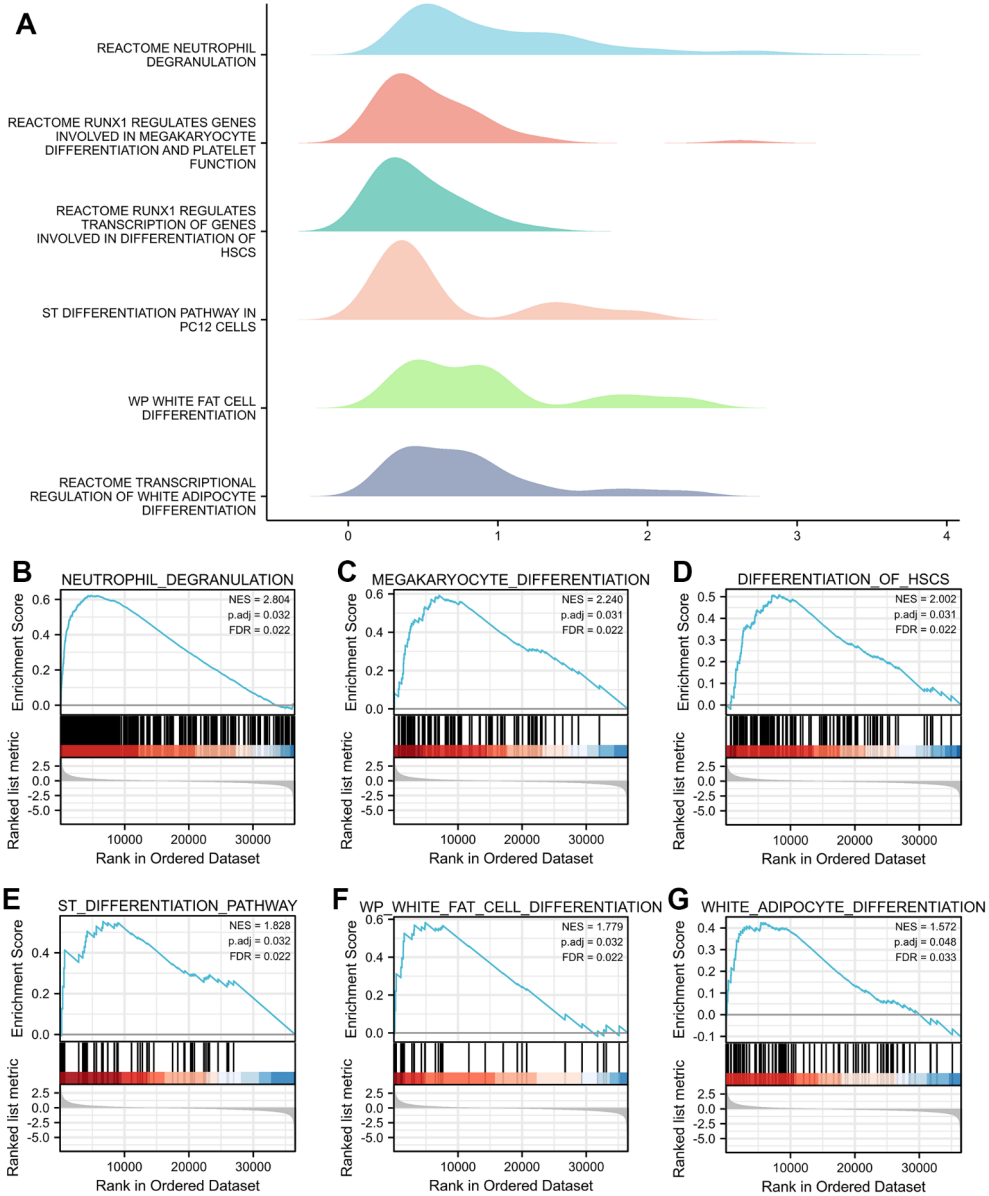
**GSEA of genes correlated with TNFAIP2 in AML patients.** (**A**) The ridge plot of the GSEA results of TNFAIP2-correlated genes revealed associations with multiple cell differentiation pathways. (**B**–**G**) Neutrophil, Megakaryocyte, Haematopoietic Stem Cell, Neural Stem Cell, White Fat Cell and White Adipocyte.

GO enrichment analyses demonstrated that TNFAIP2-correlated genes where enriched in multiple items, such as neutrophil-mediated immunity, neutrophil activation, neutrophil degranulation, ficolin-1-rich granule, and phosphatidylinositol phosphate binding (Figure 2A–2C). Additionally, the TNFAIP2-correlated genes were markedly enriched in pathways such as Tuberculosis, Osteoclast differentiation, Lysosome, Phagosome, and B-cell receptor signaling (Figure 2D). Subsequently, the results of GSEA revealed that several differentiation-associated events were enriched in the group with high expression of TNFAIP2-correlated genes, including Neutrophil, Megakaryocyte, Haematopoietic Stem Cell, Neural Stem Cell, White Adipocyte and so on. These results demonstrated a high expression of genes relevant to TNFAIP2 was involved in the increased haematopoietic differentiation in AML (Figure 3A–3G).

### Relationships between TNFAIP2 expression and immune infiltration

**Figure 4:**
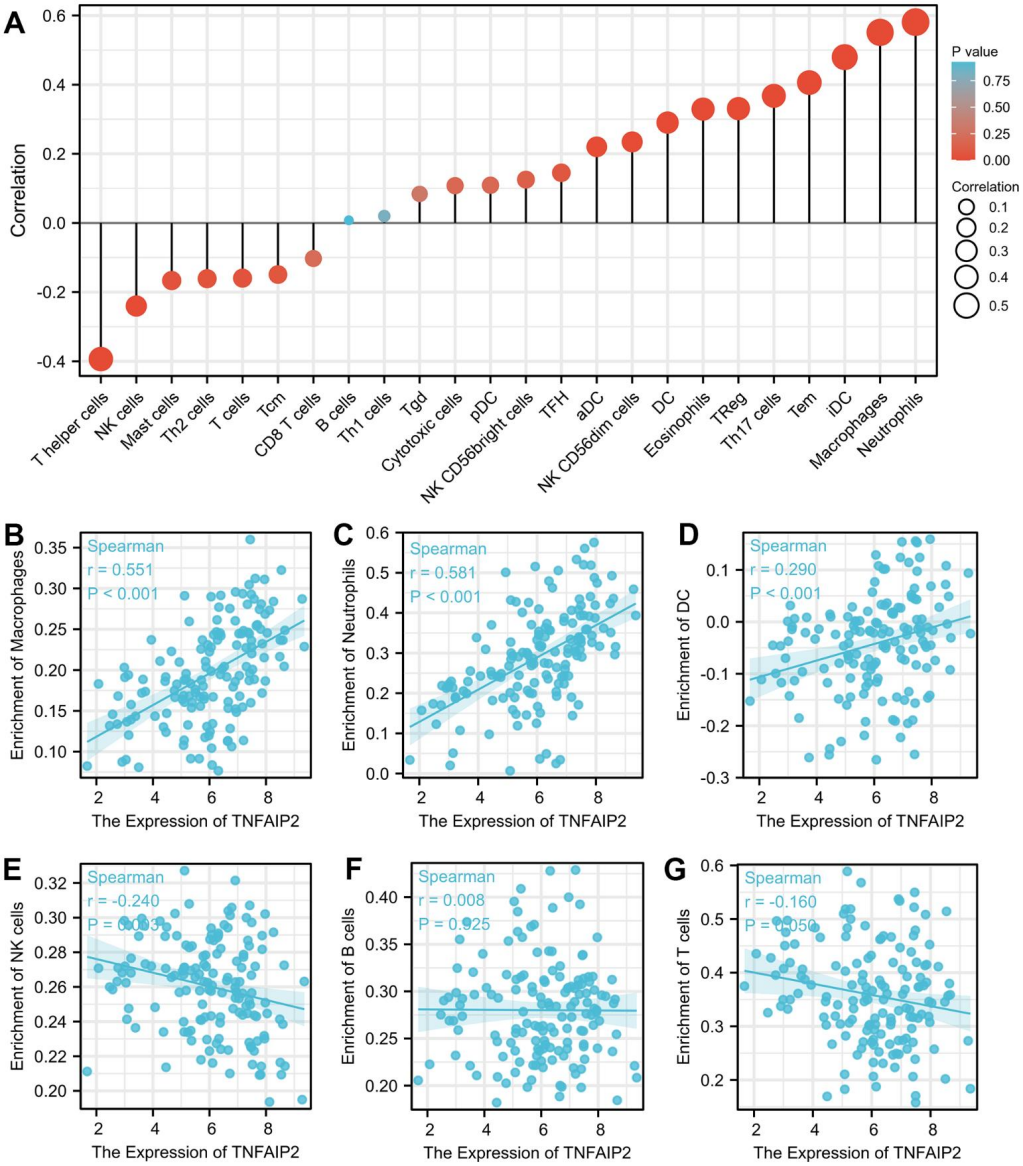
**The expression of TNFAIP2 was associated with immune infiltration in the AML microenvironment.** (**A**) Correlations between TNFAIP2 expression and the relative abundances of 24 types of immune cells. The size of the dot corresponds to the absolute Spearman correlation coefficient. (**B**–**G**) The relationships between the expression of TNFAIP2 and the relative enrichment scores of immune cells (including macrophages, neutrophils, dendritic cells, natural killer cells, B cells and T cells).

High expression of TNFAIP2 was markedly positively associated with the infiltration degree of macrophages (r = 0.551, *P* < 0.001), neutrophils (r = 0.581, *P* < 0.001), and dendritic cells (r = 0.290, *P* < 0.001). Moreover, the infiltration degree of natural killer cells and T cells in the TNFAIP2 high expression group were significantly lower than those in the TNFAIP2 low expression group (all *P* < 0.05) (Figures 4A–4G). TNFAIP2 may involve in the tumour immunomodulation, thus playing a role in inducing cell differentiation and other biological functions.

### Establishment of the risk score system

**Figure 5:**
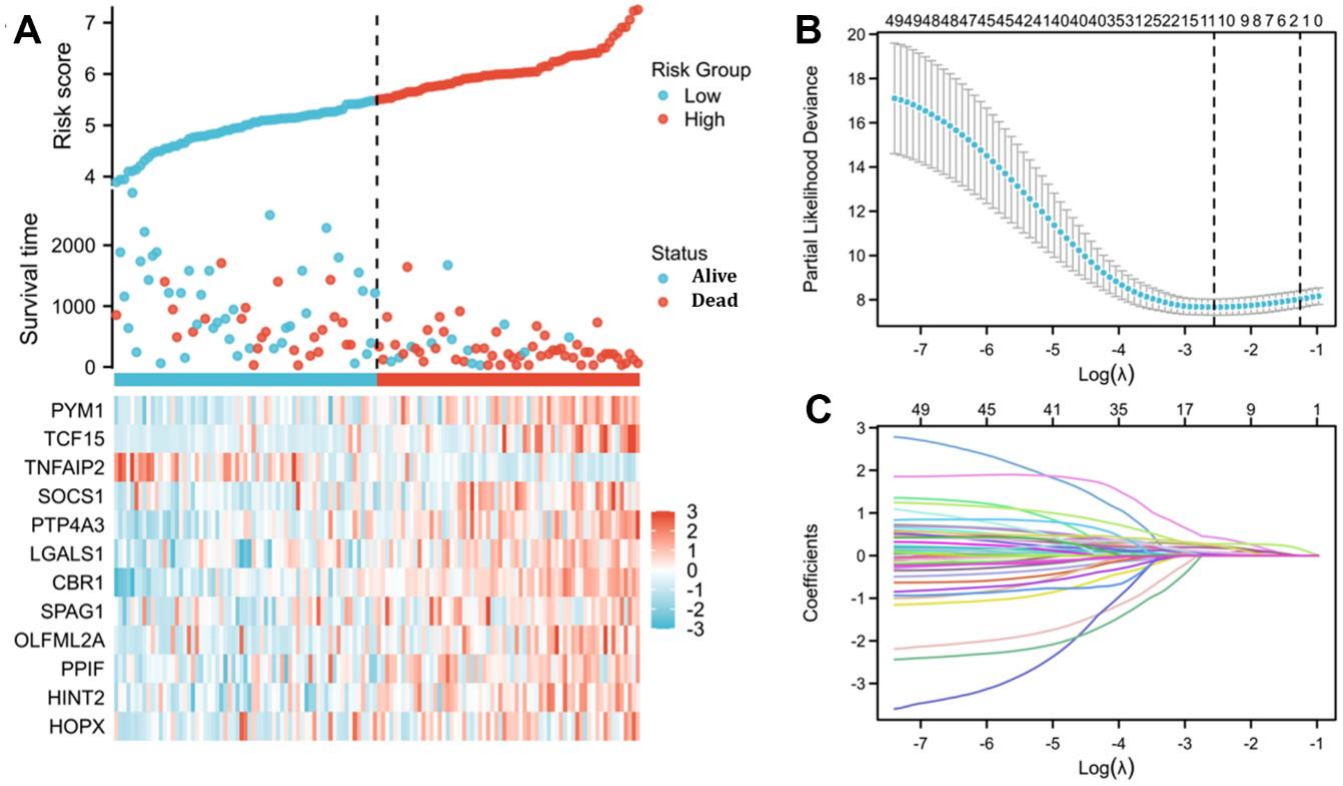
**Risk score analysis and LASSO regression based on TNFAIP2-correlated genes by Cox regression analysis in patients with AML.** (**A**) Risk score and survival time distributions and heatmaps of the expression levels of TNFAIP2-correlated genes in the TCGA database. (**B**) Cross-validation for tuning parameter screening for LASSO regression. (**C**) LASSO coefficient profiles.

In total, 12 genes, including TNFAIP2, were identified to be correlated with the prognosis of 150 AML cases (the patients’ features are listed in Supplementary Table 2). On the basis of 12 prognostic genes, risk scores were calculated and categorised into different risk arms depending on the median scoring. The risk score was distributed among the 150 patients as shown in Figure 5A. Moreover, survival time distributions suggested the possibility that there was a positive correlation between higher risk scores and worse outcome. Subsequently, the least absolute shrinkage and selection operator (LASSO) regression algorithm was used to refine the gene sets by calculating regression coefficients (Figure 5B, 5C).

### The expression of TNFAIP2 is upregulated upon acute myeloid leukaemia cell differentiation

**Figure 6:**
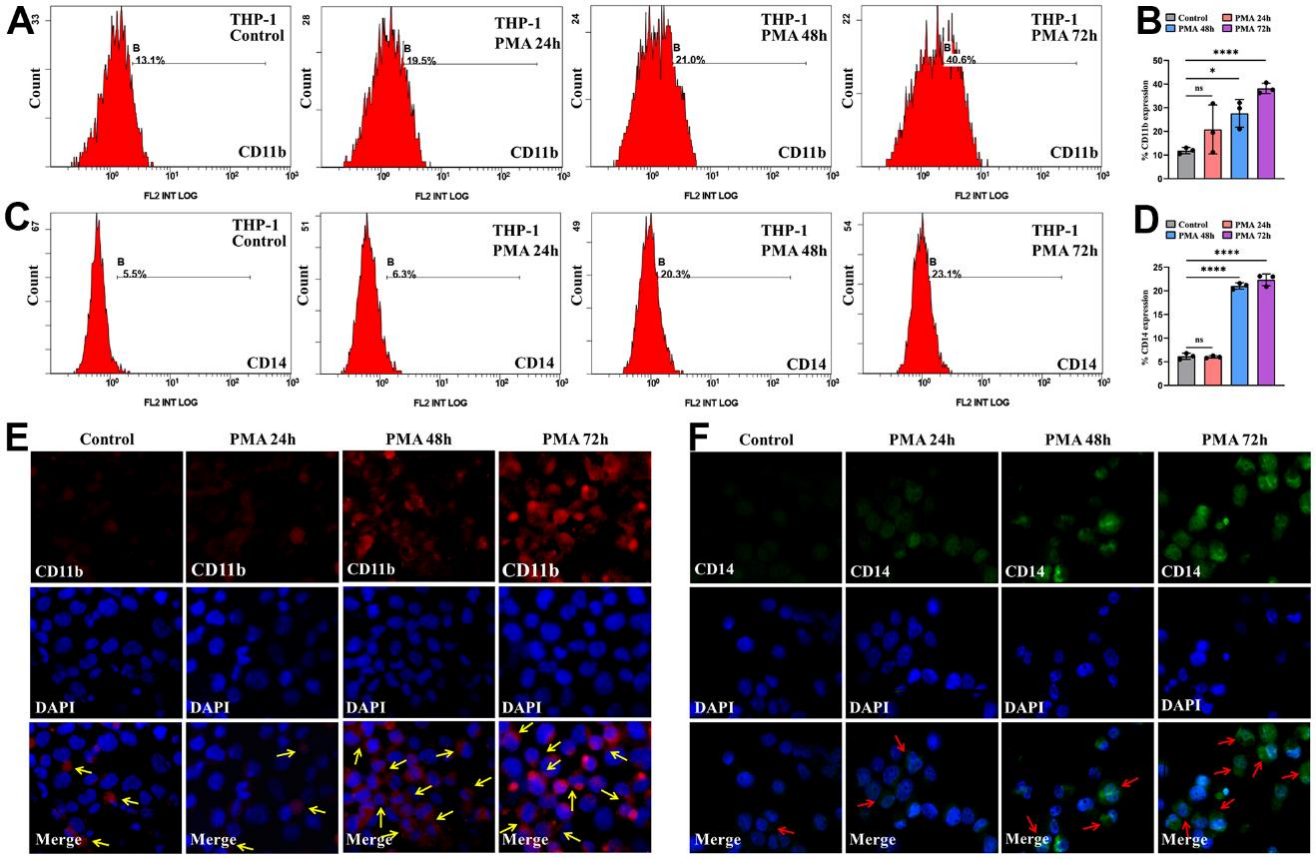
**PMA induced THP-1 cell differentiation.** (**A**) Flow cytometric determination of the CD11b+ THP-1 cell proportion. (**B**) The CD11b statistical histogram. (**C**) Flow cytometric determination of the CD14+ THP-1 cell proportion. (**D**) The CD14 statistical histogram. (**E**) CD11b and (**F**) CD14 immunofluorescence intensity in THP-1 cells. Normal distribution, ANOVA test, * *P* < 0.05, **** *P* < 0.0001.

**Figure 7:**
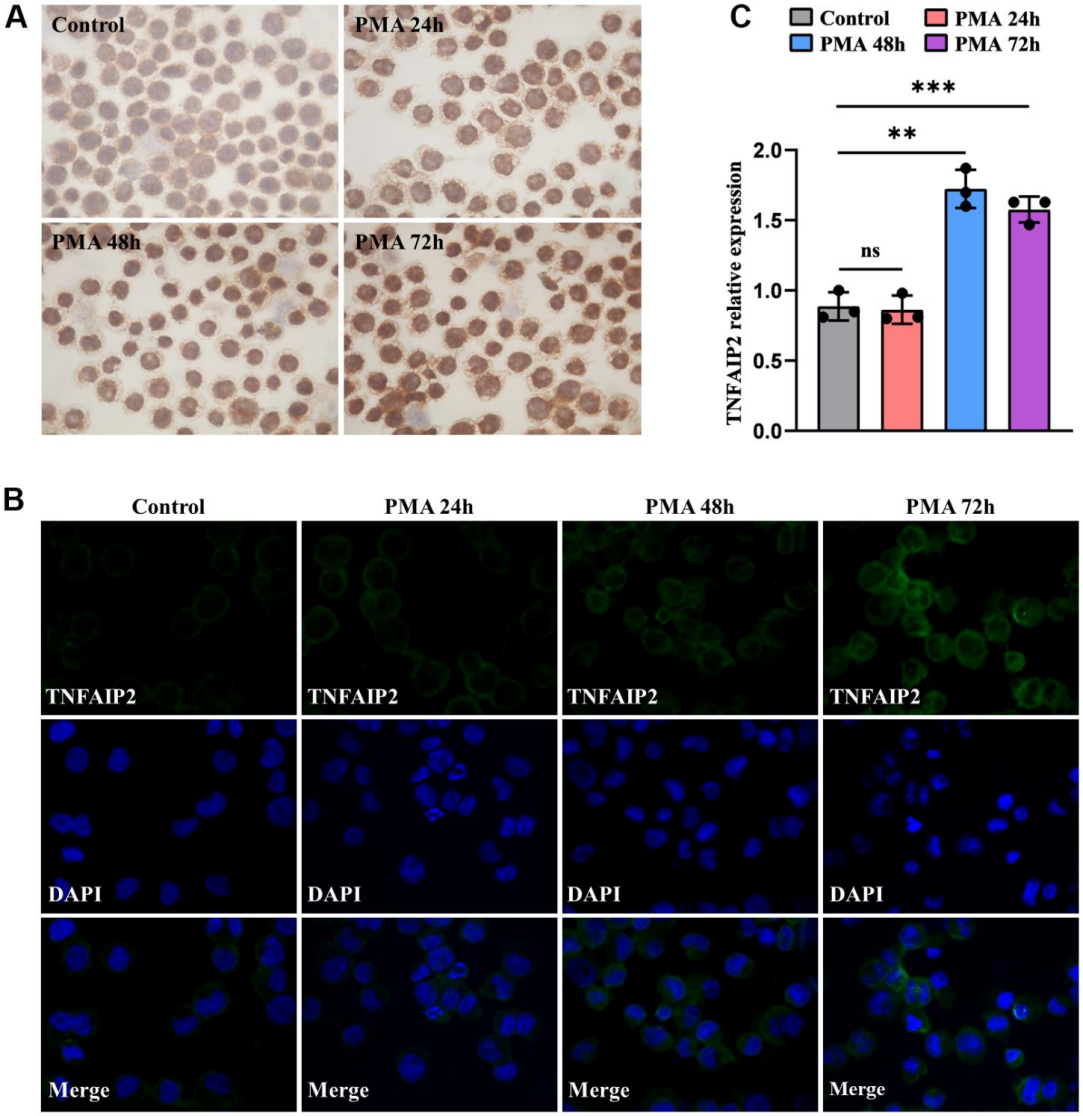
**The expression levels of TNFAIP2 mRNA and protein were increased upon monocytic leukaemia cell differentiation induced by PMA.** (**A**) Immunocytochemistry was used to evaluate TNFAIP2 protein expression in MOLM-13 cells. (**B**) The immunofluorescence intensity of TNFAIP2 protein in THP-1 cells. (**C**) qRT-PCR analysis of TNFAIP2 mRNA expression in THP-1 cells. Normal distribution, ANOVA test, ** *P* < 0.01, *** *P* < 0.001.

For exploring the association as TNFAIP2 expression and AML cell differentiation, THP-1 cells processed with 50 ng/mL PMA over 24-72 hours, and the proportions of cells positive for the differentiation-related cell surface antigens and TNFAIP2 were determined. After PMA treatment, the CD11b+ and CD14+ THP-1 cell frequencies were significantly elevated versus those in the controlled arm (*P* < 0.05, Figure 6A–6D). Similarly, the immunofluorescence intensity was increased for CD11b and CD14. (Figure 6E, 6F). Moreover, TNFAIP2 protein and mRNA expression were significantly elevated in PMA-treated THP-1 cells compared to controls (*P* < 0.05, Figure 7B, 7C). In addition, the expression level of TNFAIP2 protein was also increased in MOLM-13 cells treated with PMA (Figure 7A). These findings indicated that TNFAIP2 expression was upregulated upon cell differentiation.

### TNFAIP2 induces the differentiation of AML cells

**Figure 8:**
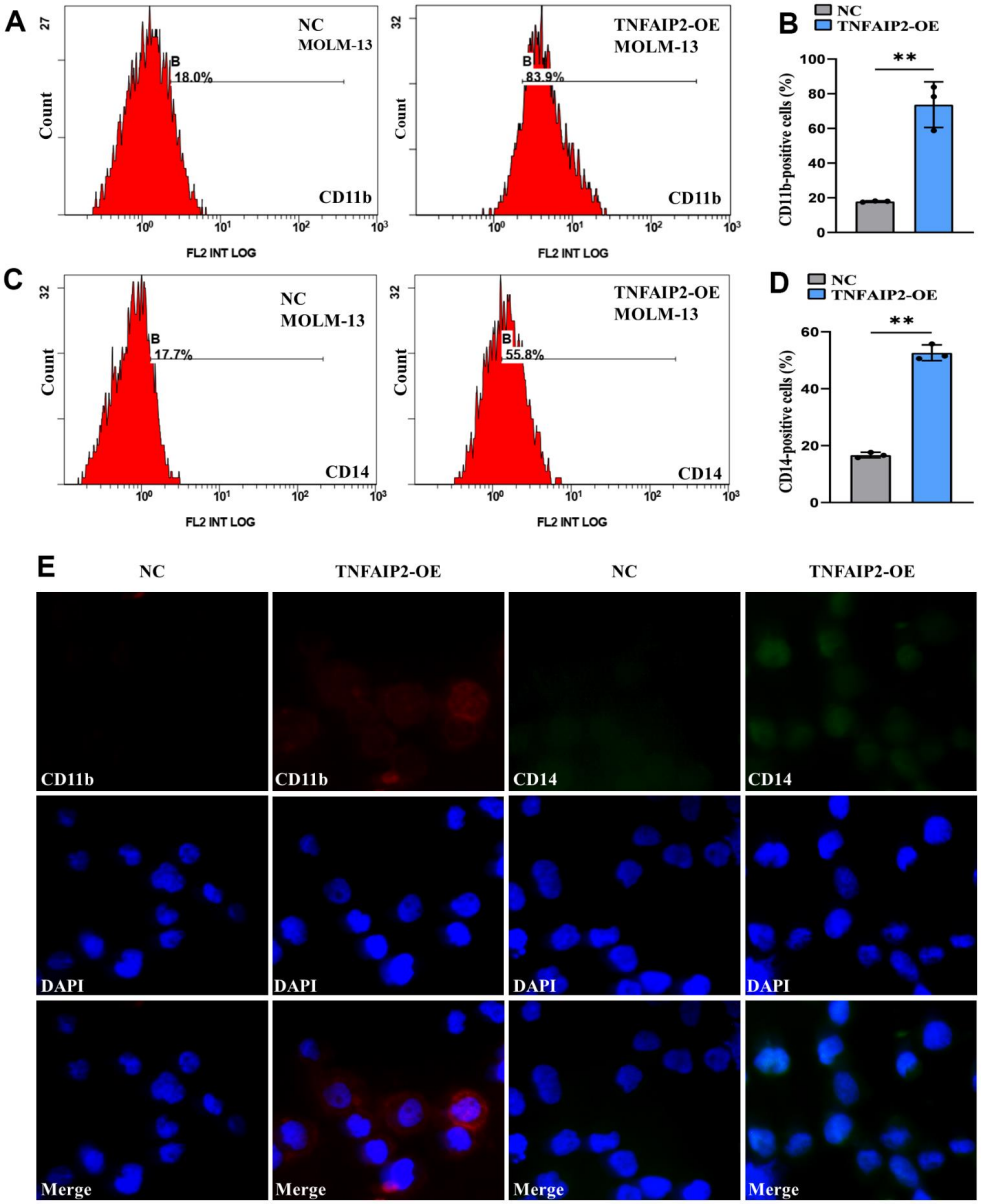
**Forced expression of TNFAIP2 significantly induced the differentiation of MOLM-13 cells.** (**A**) Flow cytometric determination of the CD11b+ cell proportion in transduced MOLM-13 cells. (**B**) The CD11b statistical histogram. (**C**) Flow cytometric determination of the CD14+ cell proportion in transduced MOLM-13 cells. (**D**) The CD14 statistical histogram. (**E**) The immunofluorescence intensity of CD11b and CD14 in transduced MOLM-13 cells. Normal distribution, t test, ** *P* < 0.01.

To prove whether TNFAIP2 can induce the differentiation of monocytic leukaemia cells, we transduced MOLM-13 cells with the TNFAIP2 overexpression (TNFAIP2-OE) construct and observed the expression of CD11b and CD14. qRT-PCR assay demonstrated that expressed TNFAIP2 mRNA was markedly upregulated in a TNFAIP2-OE arm versus a negative control arm (*P* < 0.05, Supplementary Figure 1), which indicated that TNFAIP2-OE successfully transfected MOLM-13 cells. Flow cytometry results revealed that the CD11b+ and CD14+ cell frequencies were significantly increased in MOLM-13 cells transduced with the TNFAIP2-OE construct versus a negative control arm (*P* < 0.05, Figure 8A–8D). In addition, the immunofluorescence intensities of CD11b and CD14 in a TNFAIP2-OE arm were higher than those in a negative control arm (Figure 8E). These results indicated that TNFAIP2 can induce AML cell differentiation.

### miR-146b-3p directly targets TNFAIP2 mRNA

**Figure 9:**
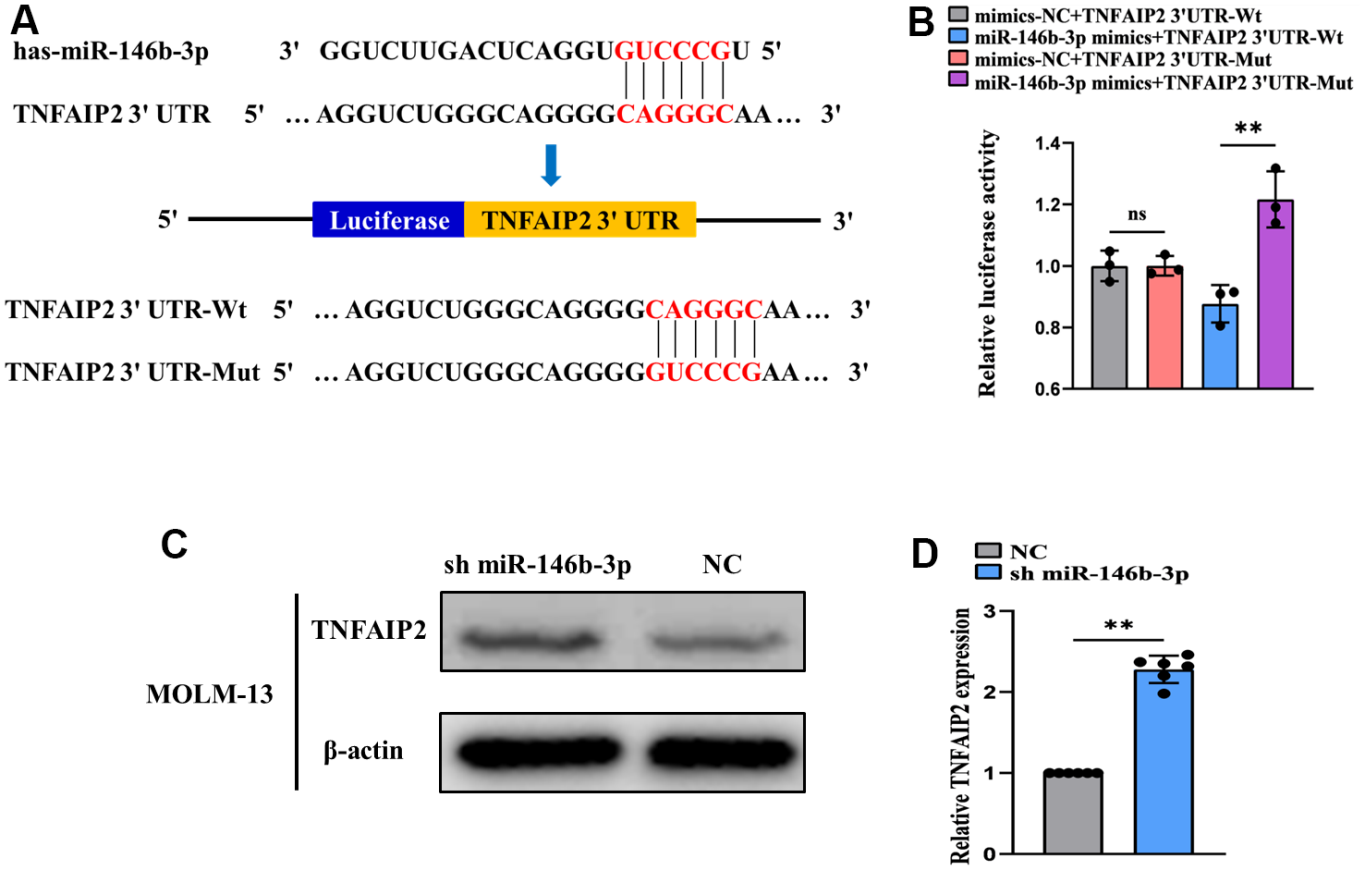
**miR-146b-3p directly targets TNFAIP2.** (**A**) TargetScan prediction of possible binding sites between miR-146b-3p and TNFAIP2. (**B**) A dual-luciferase reporter assay was used to measure luciferase activity (normal distribution, t test). (**C**) Western blot analysis of TNFAIP2 protein expression in transfected MOLM-13 cells. (**D**) qRT-PCR detection of TNFAIP2 mRNA expression in transfected MOLM-13 cells (non-normal distribution, nonparametric test Mann-Whitney). ** *P* < 0.01.

Using the TargetScan bioinformatics prediction database, we found that many miRNAs can directly target TNFAIP2. In addition, some miRNAs were downregulated upon THP-1 cell differentiation (data not shown). We determined the overlap between these two gene sets and identified miR-146b-3p. Using the TargetScan database, we predicted that bases 2~7 at the 5’ end of miR-146b-3p are complementary to the 3’UTR of TNFAIP2 mRNA (Figure 9A). To verify the direct binding of miR-146b-3p to the 3’UTR of TNFAIP2, we generated luciferase constructs containing the potential miR-146b-3p binding site in the 3’UTR of TNFAIP2 and a mutant version of the binding site (Figure 9A). The result indicated that the luciferase activity was distinctly suppressed in the miR-146b-3p mimic+TNFAIP2 3'UTR-Wt group compared with the miR-146b-3p mimic+TNFAIP2 3’UTR-Mut group (Figure 9B). These results confirmed that miR-146b-3p directly binds to the TNFAIP2 3’UTR. In addition, the expression of TNFAIP2 was upregulated in MOLM-13 cells transduced with sh miR-146b-3p vs. control group (*P* < 0.05, Figure 9C, 9D). These results demonstrated that inhibiting miR-146b-3p can attenuate its inhibitory effect on TNFAIP2 expression.

### Inhibition of miR-146b-3p induces AML cell differentiation

**Figure 10:**
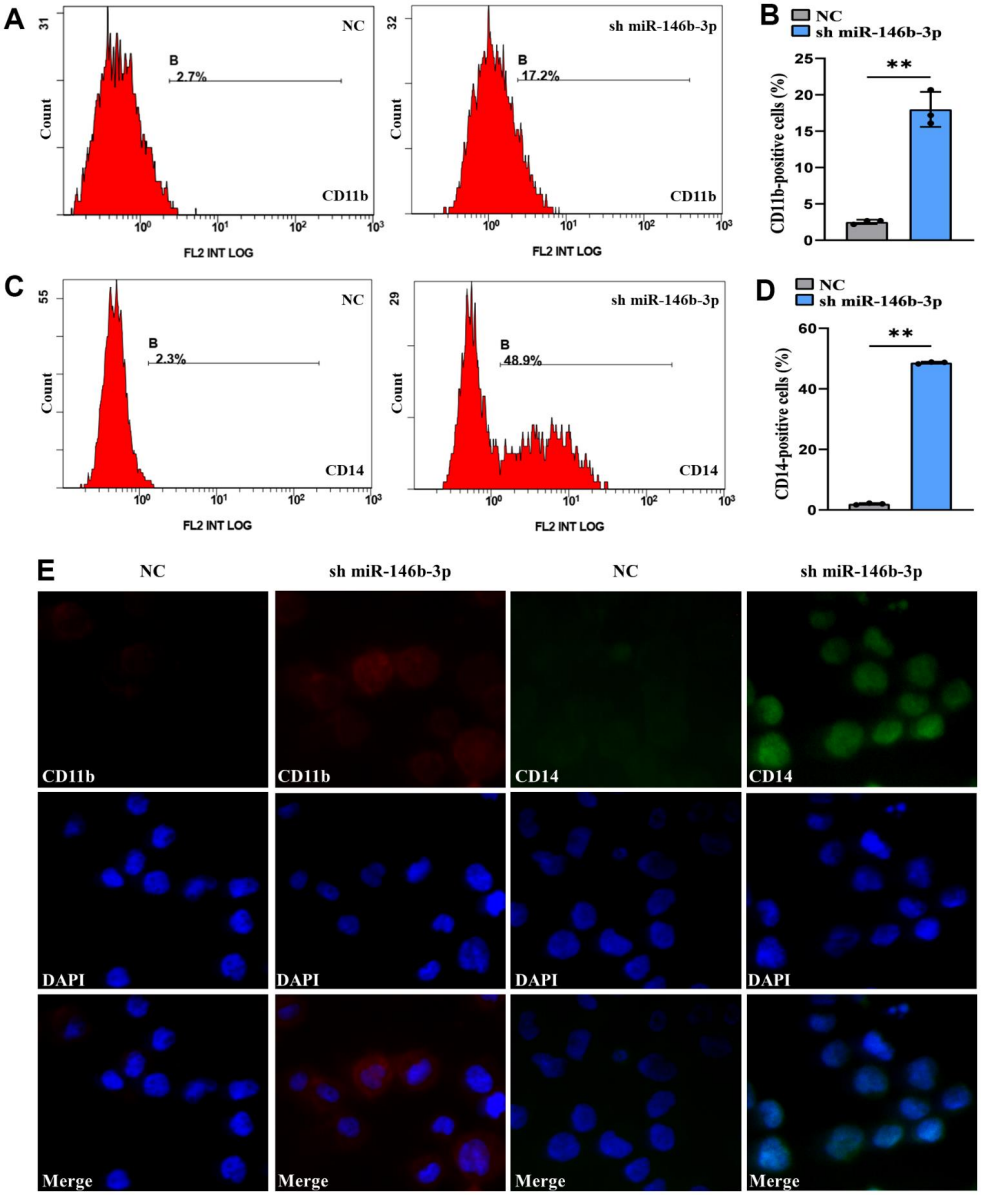
**Knockdown of miR-146b-3p significantly induced the differentiation of MOLM-13 cells.** (**A**) Flow cytometric determination of the CD11b+ cell proportion in transduced MOLM-13 cells. (**B**) The CD11b statistical histogram. (**C**) Flow cytometric determination of the CD14+ cell proportion in transduced MOLM-13 cells. (**D**) The CD14 statistical histogram. (**E**) The immunofluorescence intensity of CD11b and CD14 in transduced MOLM-13 cells. Normal distribution, t test, ** *P* < 0.01.

To elucidate the association as miR-146b-3p expression and cell differentiation, we transduced MOLM-13 cells with short hairpin miR-146b-3p (sh miR-146b-3p) and observed the CD11b+ and CD14+ cell frequencies. qRT-PCR assay demonstrated that expressed miR-146b-3p was markedly downregulated in a sh miR-146b-3p arm versus a negative control arm (*P* < 0.05, Supplementary Figure 2), suggesting that sh miR-146b-3p was successfully transduced into MOLM-13 cells. Flow cytometry results revealed that the CD11b+ and CD14+ cell frequencies were markedly increased in MOLM-13 cells transduced with sh miR-146b-3p versus a negative control arm (*P* < 0.05, Figure 10A–10D). Moreover, the immunofluorescence intensities of CD11b and CD14 in a sh miR-146b-3p group were higher than those in a negative control arm (Figure 10E). Therefore, these findings showed that suppression of miR-146b-3p expression can induce MOLM-13 cell differentiation. The above findings showed that overexpression of TNFAIP2 can induce the differentiation of MOLM-13 cells and that miR-146b-3p directly targets TNFAIP2. Considering the above results collectively, we proved that suppression of miR-146b-3p expression can attenuate the suppression of TNFAIP2 expression, thereby inducing the differentiation of MOLM-13 cells. This finding might be explained by the conclusion that cell differentiation is indeed related to the miR-146b-3p/TNFAIP2 axis.

## DISCUSSION

TNFAIP2 is abundantly expressed in bone marrow HSCs/HPCs and peripheral blood monocytes, and it participates in multiple biological functions, including cell differentiation [7, 8]. TNFAIP2 is highly expressed in various malignant tumours, such as nasopharyngeal carcinoma, triple-negative breast cancer, and glioma [24–26]. TNFAIP2 is positively correlated with poor prognosis in oesophageal carcinoma [27]. However, it has been reported that elevated levels of TNFAIP2 are strongly correlated with prolonged survival in multiple malignancies, including bladder urothelial carcinoma, sarcoma and skin cutaneous melanoma [7]. In this study, we found that the expression of TNFAIP2 in AML samples was obviously lower than that in normal bone marrow samples in the GSE9476 dataset, and this result was confirmed in another independent cohort in the Oncomine database. Thus, TNFAIP2 could act as a tumour suppressor gene in AML.

Accumulating evidence has revealed that TNFAIP2 participates in cell differentiation. The differentiation of pluripotent stem cells depends on TNFAIP2 expression [28]. The TNF-α signalling pathway activates VIM, which directs cell differentiation in the haematopoietic system. Recent studies proved that the VIM protein expression is upregulated upon induction of haematopoietic stem cell differentiation, but when TNFAIP2 is silenced, the expression of VIM decreased, and the differentiation process is arrested. TNFAIP2 may be involved in APL cell differentiation as a potential target gene [10, 11]. The expression of TNFAIP2 is upregulated upon U937 cell differentiation, indicating that TNFAIP2 might be relevant to the promotion of cell differentiation in haematological malignancies [10]. Although these studies have provided us with insight into the relationship between TNFAIP2 and cell differentiation, whether TNFAIP2 can directly induce AML cell differentiation has not been fully elucidated, nor has the underlying mechanism. Therefore, it is necessary to further investigate what role and probable mechanisms TNFAIP2 plays in inducing AML cell differentiation.

In this study, via the LinkedOmics database and GO/KEGG and gene set enrichment analyses, we found that TNFAIP2 and its correlated genes could be closely associated with several differentiation-related biological processes, such as Osteoclast, Neutrophil, Megakaryocyte, Haematopoietic Stem Cell, Neural Stem Cell, and White Adipocyte. In addition, elevated TNFAIP2 expression was remarkably related to a lower risk score in AML patients, and LASSO regression showed that TNFAIP2 was effective as a prognostic marker of AML. The etiopathogenesis of AML is firmly connected with differentiation arrest in haematopoietic stem cells in bone marrow; therefore, we speculated that TNFAIP2 is associated with cell differentiation in AML.

After PMA treatment, the CD11b+ and CD14+ THP-1 cell frequencies were significantly increased, and the expression levels of TNFAIP2 mRNA and protein were increased upon AML cell differentiation, demonstrating that TNFAIP2 might be related to cell differentiation in AML. Moreover, forced expression of TNFAIP2 increased the frequencies of CD11b+ and CD14+ MOLM-13 cells. Our results indicated that TNFAIP2 might directly induce AML cell differentiation, constituting one of the mechanisms by which PMA induces the differentiation of AML cells.

To investigate the upstream mechanism of TNFAIP2 induced AML cell differentiation, Targetscan was used to predict the upstream miRNAs interacting with TNFAIP2. In addition, some miRNAs were downregulated upon monocytic leukaemia cell differentiation. We determined the overlap between these two gene sets and identified miR-146b-3p. A miRNA binds to the 3’ untranslated region (3’UTR) of a target mRNA, resulting in decreased translation of the protein encoded by the target gene through inhibition of target gene expression or posttranslational modification. miR-146b-3p targets NF2, MAP3K10, HPGD, FAM107A, etc. mRNAs, resulting in tumorigenesis and progression in multiple malignant diseases [18–21]. To verify the direct binding of miR-146b-3p to the 3’UTR of TNFAIP2, we generated luciferase constructs containing the potential miR-146b-3p binding site in the 3’UTR of TNFAIP2 and a mutant version of the binding site. The result confirmed that miR-146b-3p directly binds to the TNFAIP2 3’UTR. In addition, the expression of TNFAIP2 was upregulated in MOLM-13 cells transduced with sh miR-146b-3p vs. control group. These results demonstrated that inhibiting miR-146b-3p can attenuate its inhibitory effect on TNFAIP2 expression. To our knowledge, there are very few reports on miR-146b-3p targeting TNFAIP2. These findings provide the basis for the following study that miR-146b-3 inhibits AML cell differentiation by targeting TNFAIP2.

Accumulating evidence suggests that miR-146b-3p participates in cell differentiation through the PI3K/AKT and PAX8/NIS pathways 22, 23. In this study, it is predicted that miR-146b-3p directly targeted TNFAIP2, and the dual-luciferase reporter assay results further verified that miR-146b-3p bound to the 3’UTR of TNFAIP2, thereby inhibiting the expression of TNFAIP2. In addition, interference with miR-146b-3p expression significantly upregulated the expression of TNFAIP2 and elevated the CD11b+ and CD14+ cell frequencies in MOLM-13 cells. Our findings indicated that TNFAIP2 might directly induce cell differentiation. It was shown that interference with miR-146b-3p expression could alleviate the inhibitory effect of TNFAIP2, thereby inducing differentiation in MOLM-13 cells. In summary, we demonstrated that TNFAIP2 is a critical driver in inducing differentiation and that the miR-146b-3p/TNFAIP2 axis involves in regulating cell differentiation in AML.

## CONCLUSIONS

Taken together, this study demonstrates that TNFAIP2 is an important modulator of AML cell differentiation. The expression of TNFAIP2 in AML samples was obviously lower than that in normal bone marrow samples. TNFAIP2 and its correlated genes were enriched in several differentiation-related pathways. Elevated TNFAIP2 expression was remarkably relevant to a lower risk score in AML patients, and LASSO analysis showed that TNFAIP2 was effective as a prognostic marker of AML. Furthermore, miR-146b-3p directly targets and inhibits TNFAIP2. Forced expression of TNFAIP2 or knockdown of miR-146b-3p can significantly induce AML cell differentiation; thus, we believe that the miR-146b-3p/TNFAIP2 axis is likely to be one of the potential mechanisms driving cell differentiation in AML.

## MATERIALS AND METHODS

### Differential expression analysis of TNFAIP2

The GSE9476 dataset was downloaded from the GEO database and included gene sequencing information from 26 AML patients and 10 healthy individuals. Differences in TNFAIP2 mRNA expression between AML and normal tissues were analysed using GraphPad Prism 8.0.2.

In addition, Oncomine was used to analyse differentially expressed TNFAIP2 in tumour and normal tissues with thresholds of |log2 fold change (FC)| > 2 and *P*_adj_ < 0.05 [29].

### LinkedOmics identification of TNFAIP2-correlated genes

LinkedOmics (http://www.linkedomics.org/) [30] provides comprehensive multiomics data for 32 cancer types in TCGA. In our study, the Pearson correlation coefficients were calculated to analyse the correlations between the expression of TNFAIP2 and that of other genes. A *P* < 0.05 was regarded as statistically significant.

### GO/KEGG enrichment analyses

Genes correlated with TNFAIP2 based on the thresholds of |log2FC| > 2 and *P*_adj_ < 0.05 were included in the analysis. GO including BP, CC, and MF categories, as well as KEGG pathing analyses, were performed by the R package clusterProfiler (see Supplementary File 1 for the specific code) [31].

### Gene set enrichment analysis (GSEA)

The R package clusterProfiler (3.14.3) was used for GSEA [26]. The gene set was analysed with one thousand permutations per analysis. A *P*_adj_ < 0.05 and FDR q < 0.25 were considered to indicate statistical significance (see Supplementary File 2 for the specific code).

### Single-sample gene set enrichment analysis (ssGSEA)

Immune infiltration analysis based on TNFAIP2 expression was conducted by ssGSEA using the GSVA package in R (3.6.3) (see Supplementary File 3 for the specific code). A total of 24 types of infiltrating immune cells were obtained as previously described [32]. Spearman correlation analysis was used to analyse the correlations between TNFAIP2 expression and the enrichment scores of the 24 types of immune cells. The Wilcoxon rank-sum test was used to analyse the enrichment scores of the high and low TNFAIP2 expression groups.

### Risk score analysis and LASSO regression analysis

The 150 patients from TCGA were included in the analysis. Genes correlated with TNFAIP2 were used in univariate Cox regression analysis to determine significant prognostic genes, and the LASSO regression technique was then performed to identify independent prognostic genes. Univariate Cox regression analysis was implemented for TNFAIP2 and its associated genes using the “ezcox” package [33]. A *P* < 0.05 indicated statistical significance. LASSO regression was conducted by the “glmnet” package [34]. LASSO regression can improve the accuracy and interpretability of a model and eliminate the problem of collinearity between independent variables. Subsequently, prognostic genes with *P* < 0.05 in univariate Cox regression analysis were included in multivariate Cox regression analysis, and prognostic genes with *P* < 0.05 in this analysis were indicated statistical significance. The identified genes were included in a risk signature, and a risk score system was constructed based on the gene expression levels and their coefficients (see Supplementary File 4 for the specific code).

### Cell differentiation induction, miRNA sequencing, and cell transfection

MOLM-13 and THP-1 cells were induced to differentiate with 50 ng/mL PMA (Sigma-Aldrich, USA). miRNA sequencing was performed after THP-1 cell processed with PMA for 72 hours. Gene sequencing was completed by Lianchuan Biotechnology Co., Ltd (Hangzhou, China). TNFAIP2-OE lentivirus and sh miR-146b-3p lentivirus were packaged by Shanghai Genechem Co., Ltd (Shanghai, China). MOLM-13 cells (1×10^5 cells/mL) were plated in 6-well plates (2 mL per well). Four microlitres of sh miR-146b-3p or 15 μL of TNFAIP2-OE lentivirus with 80 μL of enhanced solution A was added to each well, and the solution in each well was then thoroughly mixed. Lentivirally transduced cells were incubated for 16 hours. When the transduction efficiency exceeded 70%, we performed fresh culture medium containing 1 mg/L puromycin for screening of stable clones for 7 days. Once the cell transfection was successful, we conducted subsequent experiments.

### TargetScan prediction of upstream miRNAs of TNFAIP2

TargetScan (http://www.targetscan.org/vert_71/) is a popular online database for predicting miRNA target genes. It predicts target genes by considering the complementation of 2~8 bases at the 5’ end of a miRNA with the 3’UTR of an mRNA. In our study, “*Homo sapiens*” was selected as the species type, and “TNFAIP2” was selected as the gene name module.

### Dual-luciferase reporter assay

The miR-146b-3p mimic, mimic-NC, Luciferase-TNFAIP2 3′UTR-Wt, and Luciferase-TNFAIP2 3′UTR-Mut plasmids were established by GeneChem Co., Ltd. Then, 293T cells were cotransfected with TNFAIP2 3’UTR-Wt or TNFAIP2 3’UTR-Mut along with the miR-146b-3p mimic or mimic-NC for 48 hours using Roche X-tremeGENE HP (Roche, Switzerland). The Dual-Luciferase® Reporter Assay System was used to detect the luciferase activity (Promega, USA).

### Flow cytometry

Cell differentiation was assessed using immunofluorescence staining with a PE-conjugated mouse anti-human CD11b antibody and an FITC-conjugated mouse anti-human CD14 antibody (BD Bioscience, USA). The proportion of cells positive for the cell surface differentiation antigen CD11b or CD14 was evaluated using flow cytometry. Each sample was randomly analysed, and 1×10^4 events were recorded. The cell differentiation rate was determined by DIVA software.

### Immunofluorescence

A suspension of cells from each group was centrifuged, and the supernatant was discarded. The cell density was adjusted to 5×10^5 cells/mL. Five microlitres of an anti-CD11b, anti-CD14, or anti-TNFAIP2 antibodies (Santa Cruz, USA, 1:200) were incorporated into the cells. Cells treated with the anti-CD11b or anti-CD14 antibody were incubated with 4° C in darkness by 30 minutes. Cells treated with the anti-TNFAIP2 antibody were incubated one hour under room temperature. Then, we aliquoted 100 μL of the cell suspension to prepare cell smears and sealed the samples with DAPI (Abcam, USA). The fluorescence intensity of CD11b, CD14 and TNFAIP2 was determined by fluorescence microscopy.

### Immunocytochemistry

A 100 μL cell suspension was aliquoted to prepare cell smears, and the cells were fixed. Subsequently, the cell smears were flushed thrice with PBS and blocked with 1% BSA. Then cell smears were incubated with the mouse anti-TNFAIP2 antibody (Santa Cruz, USA, 1:200) at 4° C overnight. Then the cell smears were sequentially incubated with the secondary antibody and the DAB chromogen (Gene Tech, USA). Finally, the cell smears were stained with haematoxylin (Baso, Zhuhai, China), sealed with neutral resin.

### Western blot analysis

Protein extraction was conducted by radioimmunoprecipitation (RIP) assay lysis buffer (Beyotime, China). The protein specimens were electrophoretically separated on a 10% SDS-PAGE gel. The proteins were then transported onto nitrocellulose filter membranes and detected by the corresponding antibodies. The antibodies and the corresponding dilutions used for western blotting were as follows: mouse anti-TNFAIP2 antibody (Santa Cruz, USA; 1:500) and β-actin rabbit mAb (CST, USA; 1:1000).

### Real-time fluorescence-based quantitative polymerase chain reaction (qRT-PCR)

Total RNA extraction was conducted by TRIzol (Takara, Shiga, Japan). Reverse transcription of RNA into cDNA was performed using a RevertAid First Strand cDNA Synthesis Kit (Thermo Fisher Scientific, USA). Primer Premier 5.0 was used to design the primers, which were synthesized by Sangon Biotech Co., Ltd (Shanghai, China). The sequences are presented in Supplementary Table 1. qRT-PCR was conducted by a QuantStudio 7 Flex real-time PCR system (Life Technologies, USA) with PowerUpTM SYBR Green Master Mix (Thermo Fisher Scientific, USA). Relative mRNA and miRNA expression levels were calculated by the 2^-ΔΔ CT method (ΔCT = CTtarget gene – CTcontrol gene, ΔΔCT = ΔCTtreat group − ΔCTcontrol group).

### Statistical analysis

GraphPad Prism 8.0.2 was for undertaking statistics analyses and plotting histograms. Differences in TNFAIP2 mRNA expression between AML and normal tissues were analysed using t test. A t test was implemented to assess the CD11b+ or CD14+ cell frequencies between control and transfection groups. The Mann-Whitney was implemented to analyse TNFAIP2 expression between control and transfection groups. Multiple groups of data were statistically analysed using ANOVA. A *P* < 0.05 was regarded as statistically significant.

### Data-availability statement

The data are from The National Center for Biotechnology Information (https://www.ncbi.nlm.nih.gov/gds/?term=GSE9476) and The Cancer Genome Atlas (TCGA) (https://www.cancer.gov/about-nci/organization/ccg/research/structural-genomics/tcga.

## Supplementary Material

Supplementary Figures

Supplementary Tables

Supplementary File 1

Supplementary File 2

Supplementary File 3

Supplementary File 4
