# Supplementary material for: The miR-146b-3p/TNFAIP2 axis regulates cell differentiation in acute myeloid leukaemia
Source: Aging (Albany NY). 2024 Jan 24;16(2):1496–515. doi: 10.18632/aging.205441 (PMC10866442; doi:10.18632/aging.205441)
Supplement: Supplementary Figures [file aging-16-205441-s001.pdf]

SUPPLEMENTARY FIGURES

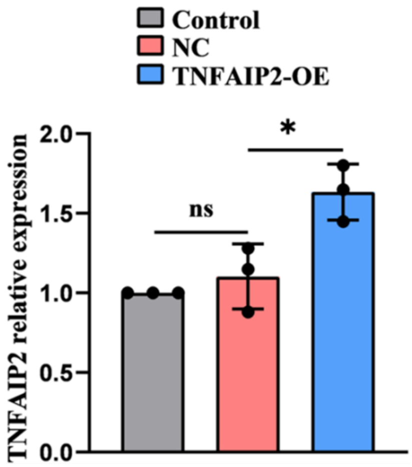

Supplementary Figure 1. MOLM-13 cells were transfected with TNFAIP2 overexpression lentivirus (TNFAIP2-OE). The expression of TNFAIP2 mRNA was significantly increased in TNFAIP2-OE group vs. NC control group. * $P < 0.05$.

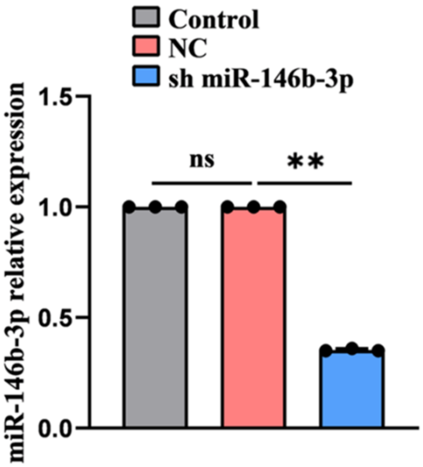

Supplementary Figure 2. MOLM-13 cells were transfected with miR-146b-3p interference lentivirus (sh miR-146b-3p). The expression of miR-146b-3p was significantly decreased in sh miR-146b-3p group vs. NC control group. ** $P < 0.01$.
