# Supplementary material for: The miR-146b-3p/TNFAIP2 axis regulates cell differentiation in acute myeloid leukaemia
Source: Aging (Albany NY). 2024 Jan 24;16(2):1496–515. doi: 10.18632/aging.205441 (PMC10866442; doi:10.18632/aging.205441)
Supplement: Supplementary Tables [file aging-16-205441-s002.pdf]

SUPPLEMENTARY TABLES

Supplementary Table 1. Representative gene primers used to perform qRT-PCR.

Gene	Primer sequences
TNFAIP2	Forward: CATATAGGGAGGAGGAAGAG
	Reverse: AAGACGCAGAACACATTG
β-actin	Forward: CATGTACGTTGCTATCCAGGC
	Reverse: CTCCTTAATGTCACGCACGAT
hsa-miR-146b-3p	Forward: CGGCCCTGTGGACTCAGT
	Reverse: AGTGCAGGGTCCGAGGTATT
	Stem-loop Primer: GTCGTATCCAGTGCAGGGTCCGAGGTATTCGCACTGGATACGACACCAGA
hsa-U6	Forward: AGAGAAGATTAGCATGGCCCCTG
	Reverse: CAGTGCAGGGTCCGAGGT
	Stem-loop Primer: GTCGTATCCAGTGCAGGGTCCGAGGTATTCGCACTGGATACGACAAAATA

Supplementary Table 2. Patients' clinical characteristics.

Characteristics	n	%
Gender		
Female	69	46.0%
Male	81	54.0%
Age		
<= 60	87	58.0%
> 60	63	42.0%
Cytogenetics		
Normal	76	50.6%
Monosomal	51	34.0%
Complex	23	15.4%
Cytogenetic risk		
Favorable	28	18.7%
Intermediate	89	59.3%
Poor	33	22.0%
FLT3 mutation		
Negative	102	68.0%
Positive	48	32.0%
NPM1 mutation		
Negative	112	74.7%
Positive	38	25.3%
OS event		
Alive	52	34.7%
Dead	98	65.3%
