# Supplementary material for: The miR-146b-3p/TNFAIP2 axis regulates cell differentiation in acute myeloid leukaemia
Source: Aging (Albany NY). 2024 Jan 24;16(2):1496–515. doi: 10.18632/aging.205441 (PMC10866442; doi:10.18632/aging.205441)
Supplement: Supplementary File 1 [file aging-16-205441-s003.docx]

**Supplementary File 1.** **The R language codes for KEGG pathway enrichment analysis.**

rm(list = ls())

a <- s$...1

library(clusterProfiler)

library(hgu133plus2.db)

z <- bitr(geneID = a,fromType = 'SYMBOL',

toType = c('ENTREZID','ENSEMBL','SYMBOL'),

OrgDb = "org.Hs.eg.db")

go<- enrichGO(z$ENTREZID,

OrgDb = org.Hs.eg.db,

ont='ALL',

pAdjustMethod = 'BH',

pvalueCutoff = 0.05,

qvalueCutoff = 0.2,

keyType = 'ENTREZID')

kegg<- enrichKEGG(z$ENTREZID,

organism = 'hsa',

keyType = 'kegg',

pvalueCutoff = 0.05,

pAdjustMethod = 'BH',

minGSSize = 3,

maxGSSize = 500,

qvalueCutoff = 0.2,

use_internal_data = FALSE)

dotplot(kegg, showCategory=20)

barplot(kegg,showCategory=20,drop=T)

cnetplot(kegg, foldChange=a)

heatplot(kegg)

library(ggplot2)
library(patchwork)
kegg<- kegg@result[kegg@result$pvalue < 0.05,]
p<-
ggplot(kegg,aes(x=GeneRatio,y=Description,colour=-log10(pvalue),size=Count))+ge
om_point()+
scale_color_gradient(low="green",high="red")+labs(y='')+theme_bw()+theme(panel.
grid = element_blank())
p B
P <- go@result[go@result$ONTOLOGY=='BP',]
BP1 <- BP[1:15,]
p1<-
ggplot(BP1,aes(x=GeneRatio,y=Description,colour=-log10(pvalue),size=Count))+geo
m_point()+
scale_color_gradient(low="green",high="red")+labs(y='')+theme_bw()+theme(panel.
grid = element_blank())
p1
cc <- go@result[go@result$ONTOLOGY=='CC',]
p2<-
ggplot(cc,aes(x=GeneRatio,y=Description,colour=-log10(pvalue),size=Count))+geom
_point()+
scale_color_gradient(low="green",high="red")+labs(y='')+theme_bw()+theme(panel.
grid = element_blank())
p2
write.csv(go@result,file = 'BP&CC.csv')
write.csv(kegg,file = 'KEGGpathway.csv')
