# Supplementary material for: The miR-146b-3p/TNFAIP2 axis regulates cell differentiation in acute myeloid leukaemia
Source: Aging (Albany NY). 2024 Jan 24;16(2):1496–515. doi: 10.18632/aging.205441 (PMC10866442; doi:10.18632/aging.205441)
Supplement: Supplementary File 2 [file aging-16-205441-s004.docx]

**Supplementary File 2. The R language codes for GSEA pathway enrichment analysis.**

library(DOSE)

library(GOSemSim)

library(clusterProfiler)

library(org.Hs.eg.db)

library(org.Mm.eg.db)

library(org.Rn.eg.db)

library(dplyr)

library(GO.db)

#

get_GO_data <- function(OrgDb, ont, keytype) {

GO_Env <- get_GO_Env()

use_cached <- FALSE

if (exists("organism", envir=GO_Env, inherits=FALSE) &&

exists("keytype", envir=GO_Env, inherits=FALSE)) {

org <- get("organism", envir=GO_Env)

kt <- get("keytype", envir=GO_Env)

if (org == DOSE:::get_organism(OrgDb) &&

keytype == kt &&

exists("goAnno", envir=GO_Env, inherits=FALSE)) {

## https://github.com/GuangchuangYu/clusterProfiler/issues/182

## && exists("GO2TERM", envir=GO_Env, inherits=FALSE)){

use_cached <- TRUE

}

}

if (use_cached) {

goAnno <- get("goAnno", envir=GO_Env)

} else {

OrgDb <- GOSemSim:::load_OrgDb(OrgDb)

kt <- keytypes(OrgDb)

if (! keytype %in% kt) {

stop("keytype is not supported...")

}

kk <- keys(OrgDb, keytype=keytype)

goAnno <- suppressMessages(

AnnotationDbi::select(OrgDb, keys=kk, keytype=keytype,

columns=c("GOALL", "ONTOLOGYALL")))

goAnno <- unique(goAnno[!is.na(goAnno$GOALL), ])

assign("goAnno", goAnno, envir=GO_Env)

assign("keytype", keytype, envir=GO_Env)

assign("organism", DOSE:::get_organism(OrgDb), envir=GO_Env)

}

if (ont == "ALL") {

GO2GENE <- unique(goAnno[, c(2,1)])

} else {

GO2GENE <- unique(goAnno[goAnno$ONTOLOGYALL == ont, c(2,1)])

}

GO_DATA <- DOSE:::build_Anno(GO2GENE, get_GO2TERM_table())

goOnt.df <- goAnno[, c("GOALL", "ONTOLOGYALL")] %>% unique

goOnt <- goOnt.df[,2]

names(goOnt) <- goOnt.df[,1]

assign("GO2ONT", goOnt, envir=GO_DATA)

return(GO_DATA)

}

get_GO_Env <- function () {

if (!exists(".GO_clusterProfiler_Env", envir = .GlobalEnv)) {

pos <- 1

envir <- as.environment(pos)

assign(".GO_clusterProfiler_Env", new.env(), envir=envir)

}

get(".GO_clusterProfiler_Env", envir = .GlobalEnv)

}

get_GO2TERM_table <- function() {

GOTERM.df <- get_GOTERM()

GOTERM.df[, c("go_id", "Term")] %>% unique

}

get_GOTERM <- function() {

pos <- 1

envir <- as.environment(pos)

if (!exists(".GOTERM_Env", envir=envir)) {

assign(".GOTERM_Env", new.env(), envir)

}

GOTERM_Env <- get(".GOTERM_Env", envir = envir)

if (exists("GOTERM.df", envir = GOTERM_Env)) {

GOTERM.df <- get("GOTERM.df", envir=GOTERM_Env)

} else {

GOTERM.df <- toTable(GOTERM)

assign("GOTERM.df", GOTERM.df, envir = GOTERM_Env)

}

return(GOTERM.df)

}
