# Supplementary material for: The miR-146b-3p/TNFAIP2 axis regulates cell differentiation in acute myeloid leukaemia
Source: Aging (Albany NY). 2024 Jan 24;16(2):1496–515. doi: 10.18632/aging.205441 (PMC10866442; doi:10.18632/aging.205441)
Supplement: Supplementary File 3 [file aging-16-205441-s005.docx]

**Supplementary File 3. The R language codes for immune infiltration analysis.**

# library(tidyverse)

library(GSVA)

library(clusterProfiler)

library(org.Hs.eg.db)

library(data.table)

library(rtracklayer)

### ssGSEA ######

## table S1 - https://doi.org/10.1016/j.immuni.2013.10.003

## pdf -> table -> read

immunity <- read.csv("~/immunity-cell-gene.csv", header = T)

# CellType AffymetrixID Symbol Gene.Symbol ENTREZ_GENE_ID

# 1 aDC 205569_at LAMP3 LAMP3 27074

# 2 aDC 207533_at CCL1 CCL1 6346

# 3 aDC 210029_at INDO IDO1 3620

# 4 aDC 218400_at OAS3 OAS3 4940

# 5 aDC 219424_at EBI3 EBI3 10148

# 6 B cells 204836_at GLDC GLDC 2731

idx <- !immunity$CellType %in% c("Blood vessels", "Normal mucosa", "SW480 cancer cells", "Lymph vessels")

immunity <- immunity[idx,]

immunity <- immunity %>%

split(., .$CellType) %>%

lapply(., function(x)(x$ENTREZ_GENE_ID))

immunity <- lapply(immunity, unique)

## Ensembl download

anno <- import('~/Homo_sapiens.GRCh38.101.gtf')

anno <- as.data.frame(anno)

anno <- anno[!duplicated(anno$gene_id),]

anno <- merge(anno, gene_symbol, by = "gene_name")

anno <- rbind(anno, data.frame(gene_name = c("KIAA1324", "IGHA1"),

gene_id = c("ENSG00000116299", "ENSG00000211895"),

ENTREZID = c("57535", "3492")))

anno <- anno[!duplicated(anno$gene_id),] ### 37417

anno <- anno[, c("gene_id", "ENTREZID")]

data <- fread("~/tpm.txt") %>%

rename("gene_id" = "V1") %>%

left_join(., anno, by = "gene_id") %>%

filter(!is.na(ENTREZID)) %>%

select(-gene_id) %>%

column_to_rownames("ENTREZID")

data <- log2(data + 1)

immu_cell <- as.data.frame(gsva(as.matrix(data), immunity, method = "ssgsea"))

### bbt plot

data <- read.table("~/file.txt", header = T)

# group aDC B cells CD8 T cells Cytotoxic cells

# 1 1.13092315 0.4709550 0.26202395 0.5944611 0.5130117

# 3 0.55644003 0.1800251 -0.07081909 0.5197230 0.2135559

# 4 0.44696904 0.3350859 0.05579749 0.5908900 0.3135561

# 5 0.05474605 0.1191767 0.02578815 0.5541712 0.2068595

# 7 0.61364297 0.1563856 0.09869185 0.5518254 0.2321028

# 8 0.41079217 0.4588979 0.50105493 0.5996277 0.4866508

data1 <- NULL

for(i in 2:25){

cor <- cor.test(data[,i], data[,1], method = "pearson")

data1 <- rbind(data1,

data.frame("group" = "a",

"cell" = colnames(data)[i],

"cor" = cor$estimate,

"p" = cor$p.value))

}

data1 <- data1[order(data1$cor),]

data1$cell <- factor(data1$cell, levels = data1$cell)

ggplot(data1, aes(x = cell, y = cor)) +

geom_segment(aes(xend=cell,yend=0)) +

geom_hline(yintercept = 0) +

geom_point(aes(col=p, fill = p, size=abs(cor))) +

coord_flip()
