# Supplementary material for: The miR-146b-3p/TNFAIP2 axis regulates cell differentiation in acute myeloid leukaemia
Source: Aging (Albany NY). 2024 Jan 24;16(2):1496–515. doi: 10.18632/aging.205441 (PMC10866442; doi:10.18632/aging.205441)
Supplement: Supplementary File 4 [file aging-16-205441-s006.docx]

**Supplementary File 4. The R language codes for Cox regression analysis.**

library(tidyverse)

library(survival)

library(readxl)

## read data

data <- read_xlsx("~/file.xlsx")

## tidy data

# data$event <- as.numeric(data$event)

# data$time <- as.numeric(data$time)

### numeric

data$Age <- as.numeric(data$Age)

data$Score <- as.numeric(data$Score)

### factor

data$Sex <- factor(data$Sex, levels = c("Male", "Female"))

data$Grade <- factor(data$Grade, levels = c("0", "1", "2"))

data$Stage <- factor(data$Stage, levels = c("Stage1", "Stage2", "Stage3", "Stage4"))

## summary

fit <- survfit(Surv(time, event) ~ Sex, data = data)

fit

# Call: survfit(formula = Surv(time, event) ~ Sex, data = data)

#

# n events median 0.95LCL 0.95UCL

# Sex=Female 90 53 426 348 550

# Sex=Male 138 112 270 212 310

## univariable Cox

fit <- coxph(Surv(time, event) ~ Sex, data = data)

summary(fit)

# Call:

# coxph(formula = Surv(time, event) ~ Sex, data = data)

#

# n= 228, number of events= 165

#

# coef exp(coef) se(coef) z Pr(>|z|)

# SexMale 0.5310 1.7007 0.1672 3.176 0.00149 **

# ---

# Signif. codes: 0

#

# exp(coef) exp(-coef) lower .95 upper .95

# SexMale 1.701 0.588 1.226 2.36

#

# Concordance= 0.579 (se = 0.021 )

# Likelihood ratio test= 10.63 on 1 df, p=0.001

# Wald test = 10.09 on 1 df, p=0.001

# Score (logrank) test = 10.33 on 1 df, p=0.001

fit <- coxph(Surv(time, event) ~ Age, data = data)

summary(fit)

# Call:

# coxph(formula = Surv(time, event) ~ Age, data = data)

#

# n= 228, number of events= 165

#

# coef exp(coef) se(coef) z Pr(>|z|)

# Age 0.019543 1.019735 0.008735 2.237 0.0253 *

# ---

# Signif. codes: 0

#

# exp(coef) exp(-coef) lower .95 upper .95

# Age 1.02 0.9806 1.002 1.037

#

# Concordance= 0.55 (se = 0.025 )

# Likelihood ratio test= 5.13 on 1 df, p=0.02

# Wald test = 5.01 on 1 df, p=0.03

# Score (logrank) test = 5.02 on 1 df, p=0.03

## multivariable Cox

fit <- coxph(Surv(time, event) ~ Age + Sex + Stage + Score, data = data)

summary(fit)

# Call:

# coxph(formula = Surv(time, event) ~ Age + Sex + Stage + Score,

# data = data)

#

# n= 224, number of events= 161

# (4 observations deleted due to missingness)

#

# coef exp(coef) se(coef) z Pr(>|z|)

# Age 0.010922 1.010982 0.008972 1.217 0.2235

# SexMale 0.536001 1.709159 0.169829 3.156 0.0016 **

# StageStage2 0.390113 1.477148 0.204289 1.910 0.0562 .

# StageStage3 0.693144 1.999993 0.283818 2.442 0.0146 *

# StageStage4 1.830114 6.234597 1.035948 1.767 0.0773 .

# Score -0.009346 0.990698 0.007127 -1.311 0.1897

# ---

# Signif. codes: 0

#

# exp(coef) exp(-coef) lower .95 upper .95

# Age 1.0110 0.9891 0.9934 1.029

# SexMale 1.7092 0.5851 1.2252 2.384

# StageStage2 1.4771 0.6770 0.9898 2.205

# StageStage3 2.0000 0.5000 1.1467 3.488

# StageStage4 6.2346 0.1604 0.8185 47.491

# Score 0.9907 1.0094 0.9770 1.005

#

# Concordance= 0.654 (se = 0.025 )

# Likelihood ratio test= 32.28 on 6 df, p=1e-05

# Wald test = 32.66 on 6 df, p=1e-05

# Score (logrank) test = 34.96 on 6 df, p=4e-06
